# Successful Treatment of Acute Lymphoblastic Leukemia in a Child with Trisomy 21 and Complex Congenital Heart Disease with Mechanical Prosthetic Valve

**DOI:** 10.1155/2012/193093

**Published:** 2012-10-02

**Authors:** Saima Alvi, Evan Shereck, Manraj K. S. Heran, George G. S. Sandor, Shahrad Rod Rassekh

**Affiliations:** ^1^Division of Pediatric Oncology, Hematology and BMT, Department of Pediatrics, British Columbia's Children's Hospital, University of British Columbia, 4480 Oak Street, Vancouver, BC, Canada V6H 3V4; ^2^Department of Pediatrics, Oregon Health & Science University, Portland, OR 97239, USA; ^3^Division of Pediatric Interventional Radiology, Children's and Women's Health Center of British Columbia, University of British Columbia, Vancouver, BC, Canada V6H 3V4; ^4^Division of Pediatric Cardiology, Department of Pediatrics, British Columbia's Children's Hospital, University of British Columbia, Vancouver, BC, Canada V6H 3V4

## Abstract

A 10-year-old girl with trisomy 21 and complex congenital heart disease presented with acute lymphoblastic leukemia. Her chemotherapy required modifications due to poor baseline cardiac status and a mechanical prosthetic heart valve that was dependent on anticoagulation. We describe our management including the use of low-molecular-weight heparin as anticoagulation for a mechanical heart valve, the safe delivery of intrathecal chemotherapy included bridging with unfractionated heparin, and the use of fluoroscopic guidance to minimize the risk of bleeding. Adjustments were made to avoid anthracyclines. The child tolerated therapy well without complications and remains relapse free five years after diagnosis.

## 1. Introduction

A 10-year-old female with trisomy 21 with a known complex cardiac history including a mechanical mitral valve presented to our hospital with a one-week history of progressive weakness, bruising, petechiae, fever, and pallor. Past history was significant for transient abnormal myelopoiesis (TAM) at birth with a peripheral blast count of 0.03 × 10^9^/L that resolved without any treatment. She had been diagnosed at birth with an atrioventricular septal defect (AVSD) and had pulmonary artery (PA) banding at the age of 2 weeks and then had corrective surgery for her AVSD at the age of 3 months. This surgery resulted in severe mitral valve insufficiency which required the placement and subsequent revision of a mechanical prosthetic mitral heart valve, requiring oral anticoagulation (OAC) with warfarin. At 7 years of age, she underwent prosthetic valve replacement; however, she developed severe mediastinitis that necessitated the replacement of this new valve. Postoperatively, she developed transient heart block and multiple episodes of bradycardia requiring the insertion of a pace-maker device. Her cardiac condition was finally stabilized, and she was followed for the next 3 years with stable cardiac function and therapeutic international normalization ratio (INR) levels on warfarin. 

At the age of 10 years, she presented with significant bruising which prompted a CBC and coagulation profile to be done. Physical examination revealed a pale, unwell child who had petechiae, bruising, and hepatosplenomegaly. Her blood counts showed hemoglobin 38 g/L, white cell count 0.6 × 10^9^/L, neutrophils 0.23 × 10^9^/L, blast count of 0.12 × 10^9^/L, and platelets <5 × 10^9^/L. The INR was significantly elevated at 6.41. She was admitted to the intensive care unit and her OAC was changed to unfractionated heparin (UFH) and the warfarin effect was reversed with vitamin K. After initial stabilization of her anticoagulation, UFH was held for four hours, and a diagnostic bone marrow biopsy and lumbar puncture were performed. The marrow confirmed a diagnosis of pre-B acute lymphoblastic leukemia (ALL) and the CSF examination was clear of disease. 

Given her complex cardiac history, decisions had to be made with regard to how to treat her leukemia. After discussion with her cardiologist, it was felt that treatment with anthracyclines would be a significant risk given her history of four open-heart procedures and decreased cardiac function. In addition, there were discussions about how to manage the delivery of intrathecal chemotherapy given that she was dependent on anticoagulation for maintenance of her prosthetic heart valve. 

Despite having just turned 10 years old a few weeks prior to diagnosis, we elected to treat as per the standard risk protocol because it does not utilize anthracyclines in induction, as opposed to the high-risk ALL protocol. She was therefore placed on a slightly modified protocol using the Children's Oncology Group Protocol AALL0331 as a backbone, with modifications made due to her cardiac history (see Table 1). We also opted to treat with augmented delayed Intensification which includes extra doses of vincristine, Peg-Asparaginase, and intrathecal methotrexate as a replacement for the removal of all anthracyclines. Leucovorin rescue was utilized for all intrathecal methotrexate doses as per the recommendations for patients with trisomy 21. We elected to give only 11 intrathecal methotrexate treatments in total based on the European BFM protocols [1], as we thought that the risk of bleeding complications with additional lumbar punctures outweighed the further benefit of these treatments.

During the initial seven months of chemotherapy, we decided to use low-molecular-weight heparin (LMWH) instead of warfarin for her anticoagulation, with anti-10a level target range of 1.25–1.5 units/mL. This higher target range was based on concerns that higher levels are required to maintain mechanical valves. Her anti-10a level during therapy ranged between 1.06 and 1.48. The LMWH was bridged with UFH before every intrathecal (IT) treatment. Furthermore, all IT chemotherapy was given under fluoroscopic guidance in order to decrease the risk of lumbar puncture-related complications, especially hemorrhage. Once all the ITs were delivered, we elected to switch her back to OAC with regular monitoring of INR by a point of care (POC) monitor, which was done on a weekly basis. Her anticoagulation was well controlled with an INR range between 1.8 and 4.4 while on chemotherapy. During chemotherapy, she had no bleeding or thrombotic complications. However, she did register a critically elevated INR of 8 a few weeks after therapy had been completed, likely due to the discontinuation of 6-mercaptopurine (6-MP). She developed brief epistaxis and was treated with a single dose of vitamin K. She did not have any episodes of bacteremia, endocarditis, or other toxicities. She completed therapy 3 years ago and is currently in remission and doing well. 

## 2. Discussion

Children with trisomy 21 have an approximately 20 times increased risk for developing acute leukemia than non-Down syndrome (DS) children. Almost 10% of DS patients are born with TAM, which is a transient self-limiting leukemia that occurs almost exclusively in newborns with DS [2]. Our patient had TAM at birth that had spontaneously resolved; however, 10 years later, she presented with ALL. Her presentation was further complicated by the presence of significant underlying cardiac history with the need for long-term anticoagulation. 

To date, there are no consensus guidelines available for bridging therapy with anticoagulants for children who have mechanical prosthetic heart valves and are undergoing invasive procedures while on anticancer treatment. Wiernikowski and Athale gathered evidence in an attempt to construct consensus guidelines entailing the use of different anticoagulation agents and the short-term reversal around the time of invasive procedures in children with cancers [3]. However these guidelines do not account for the unique circumstance where anticoagulation is critical for survival, such as in our child with a mechanical mitral valve. 

Our patient was placed on heparin-based therapy instead of warfarin during the more intensive portion of therapy due to two major factors. The first was the concern about being able to maintain therapeutic INR values given the diet changes, use of antibiotics, mucositis, and drug interactions that are all frequent issues during intensive therapy. The second reason for LMWH was the need to choose an anticoagulant that could be reversed easily to enable invasive procedures to be performed safely. Once the more intensive portion of therapy was completed and all intrathecal injections were delivered, she was then transitioned back to warfarin and the INR was monitored with a POC monitor, an effective method for home-based monitoring [4]. There are two reported trials in which LMWH has been found to be safe and efficacious in preventing thrombosis in children with cancers [5, 6]. There are no reports in the literature regarding the use of LMWH in children with mechanical valves. The bridging therapy using UFH and the use of LMWH with target anti-Xa levels that were higher than the usual therapeutic range were based on studies in pregnant females with artificial valves around the time of delivery or procedures [7–10]. The decision to use fluoroscopic guidance was based on studies that have shown a lower incidence of traumatic lumbar punctures when performed under fluoroscopic guidance compared to the bed side procedure [11]. This was indeed successful, as our patient did not have any bleeding with any of her procedures.

 This case also highlights the interaction between 6-mercaptopurine and warfarin. There are four reports in the literature of the drug interaction between 6-MP and warfarin, resulting in the requirement for higher dosing of warfarin [12–15]. Physicians should be aware that patients should have close monitoring once treatment ends to ensure the INR remains in the therapeutic range.

 The treatment regimen was based on standard-risk leukemia protocols. However, anthracyclines were removed all together from the treatment plan due to concerns regarding this child's underlying cardiac history. Anthracyclines have been thought to have an important role in the treatment of childhood ALL although there are no randomized trials in which this has been investigated [16], and the decision to avoid its use must be reserved for only the most exceptional circumstances. 

In conclusion, we were able to successfully manage a child with DS with a constellation of concerns using a labor intensive and modified approach. The child tolerated all aspects of treatment remarkably well and remains alive and relapse free 5 years after diagnosis.

## Figures and Tables

**Table 1:** Modified chemotherapy regimen used for our subject using the standard-risk ALL0331 backbone with modifications.

Chemotherapeutic drug	Route	Dose	Days
Phase i: induction			
Intrathecal ara-C	IT	70 mgs	Day 1
^ ∗^Intrathecal methotrexate	IT	15 mgs	Day 8, 29
Vincristine	IV	1.5 mgs/m^2^	Day 1, 8, 15, 22
PEG-asparaginase	IM	1500 iu/m^2^	Day 4
^ ∗∗^Prednisone	PO	60 mg/m^2^	Day 1–28
^ ∗∗^PO Prednisone as per CCG 1961 added instead of dexamethasone.			

Phase ii: standard consolidation			
^ ∗^Intrathecal methotrexate	IT	15 mgs	Day 1, 8, 15
Vincristine	IV	1.5 mgs/m^2^	Day 1
6-Mercaptopurine	PO	75 mgs/m^2^	Day 1–28

Phase iii: standard interim maintenance			
Dexamethasone	PO	6 mgs/m^2 ^	Days 1–5
			Days 29–33
Vincristine	IV	1.5 mgs/m^2^	Days 1 and 29
6-Mercaptopurine	PO	75 mgs/m^2^	Days 1–50
^ ∗^Intrathecal methotrexate	IT	15 mgs	Day 29

Phase iv: augmented delayed intensification			
Vincristine	IV	1.5 mgs/m^2^	Days 1, 8, 15, 43, 50
PEG-asparaginase	IM	1500 iu/m^2^	Days 4 or 5 or 6 and 43
Cyclophosphamide	IV	1000 mgs/m^2^	Day 29
Cytarabine	IV	75 mgs/m^2^	Days 29–32
			Days 36–39
Thioguanine	PO	60 mgs/m^2^	Days 29–42
^ ∗^Intrathecal methotrexate	IT	15 mgs	Day 8, 29, 43
Anthracyclines (doxorubicin) removed from the protocol due to the underlying cardiac dysfunction.			

Phase v: maintenance (total 8 cycles each of 84 days)			
Intrathecal methotrexate	IT	15 mgs	Day 1 of cycle 1 and 2
6-Mercaptopurine	PO	75 mgs/m^2^	Day 1–84
Methotrexate	PO	20 mgs/m^2^	weekly
Vincristine	IV	1.5 mgs/m^2^	Day 1, 29, 57
Dexamethasone	PO	6 mgs/m^2^	5 days every 4 weeks days 1–5, 29–33, 57–61

^∗^Leucovorin PO 5 mg/m^2^ every 12 hrs 2 doses 48 and 60 hrs after each IT.

Note: IT MTX doses are age adjusted. All intrathecals were administered under fluoroscopic guidance.
